# A SINE Insertion in *F8* Gene Leads to Severe Form of Hemophilia A in a Family of Rhodesian Ridgebacks

**DOI:** 10.3390/genes12020134

**Published:** 2021-01-21

**Authors:** Alexandra Kehl, Anita Haug Haaland, Ines Langbein-Detsch, Elisabeth Mueller

**Affiliations:** 1 Laboklin GmbH&Co.KG, Steubenstraße 4, 97688 Bad Kissingen, Germany; langbein@laboklin.com (I.L.-D.); mueller@laboklin.com (E.M.); 2 Department of Companion Animal Clinical Sciences, Faculty of Veterinary Medicine, Norwegian University of Life Sciences, P.O. Box 5003, 1432 Ås, Norway; anita.haug.haaland@nmbu.no

**Keywords:** dog, hemostasis disorder, mutation

## Abstract

Hemophilia A is the most common coagulation factor disorder in humans and dogs. The disease is characterized by the lack or diminished activity of Factor VIII (FVIII), caused by variants in the *F8* gene and inherited as an X chromosomal trait. Two related male Rhodesian Ridgebacks were diagnosed with Hemophilia A due to reduced FVIII activity. The purpose of the study was to determine the genetic cause and give breeding advice for the remaining family members in order to eradicate the variant. By Sanger sequencing a short interspersed nuclear element (SINE) insertion in exon 14 of the *F8* gene was found. Perfect correlation of this genetic variant with clinical signs of hemophilia A in the family tree, and the lack of this genetic variant in more than 500 unrelated dogs of the same and other breeds, confirms the hypothesis of this SINE being the underlying genetic cause of Hemophilia A in this family. The identification of clinically unaffected female carriers allows subsequent exclusion of these animals from breeding, to avoid future production of clinically affected male offspring and more subclinical female carriers.

## 1. Introduction

Inherited bleeding disorders encompass aberrations in primary hemostasis, coagulation, and fibrinolysis caused by genetic variants interfering directly or indirectly with platelet function. Hemophilia A is the most common coagulation factor disorder in dogs resulting from mutations in *F8* gene [1,2]. Factor VIII (FVIII), one of the largest coagulation factors, consists of a heavy and a light chain. It is present in the blood linked to von Willebrand factor (vWF) in a non-covalent complex. In its activated form, FVIIIa acts as a cofactor for the prothrombinase complex in the intrinsic coagulation pathway by catalyzing activation of FX in the presence of FIXa [3]. As the *F8* gene lies on the X chromosome, hemophilia A is inherited in an X chromosomal recessive manner. Male dogs are phenotypically affected with just one mutated X chromosome (named hemizygotes) while female dogs are heterozygous carriers passing the mutated allele to its offspring. Hemophilia A has been recognized in dogs for decades [4], and a dog colony (Chapel Hill colony) for the comparative studies of the disease was established at the University of North Carolina at Chapel Hill, USA. Later, an intron 22 inversion similar to a genetic mutation commonly found in human patients, was identified in both the Chapel Hill colony and an unrelated dog colony at Queens Hospital, Canada [5,6]. Other breed-specific genetic variants causing Hemophilia A are later described in the breeds Old English Sheepdog [7], Boxer [8], German Shepherd [8,9], and Havanese [10].

In Rhodesian Ridgebacks, hemophilia B resulting from a genetic variant in *F9* gene is well known and genetic screening of breeding animals is common [11]. However, in the winter of 2019, an 8-week-old male Rhodesian Ridgeback was presented with hemophilia A at the University Animal Hospital at the Norwegian University of Life Sciences. A retrospective investigation into the clinical history of male siblings, followed by clinicopathological testing where possible, confirmed a second affected dog from the litter and gave clinical suspicion of a third affected dog.

The aim of the study was to identify the underlying causative *F8* gene defect to allow targeted breeding and eradication of the variant and the disease from the breed.

## 2. Materials and Methods

### 2.1. Animals and Test Material

The study animals included two privately owned Rhodesian Ridgeback breeding dogs (dam and sire), and 13 of their litter. No consanguinity of the parents or grandparents was known. One unrelated dog of the same breed and household and 20 unrelated Rhodesian Ridgebacks were used as a control group. A blood sample consisting of whole blood anticoagulated with EDTA was sent to Laboklin laboratories for routine genetic testing of other diseases. Leftover blood samples and isolated DNA were used for this study. 

### 2.2. DNA Extraction and Amplification

Genomic DNA was isolated from EDTA blood samples using MagNA Pure (Roche, Basel, Suisse) with the MagNA Pure DNA and viral NA Small kit according to manufacturer’s instructions. PCR was performed for amplification of all 26 exons containing exon-intron boundaries by using FastStart Mastermix (Roche) and automated thermal cycler. Used primers are listed in Table S1.

### 2.3. Sanger Sequencing and Analysis

Amplified DNA fragments were directly sequenced on an ABI Genetic Analyser 3130 (Life Technologies, Carlsbad, CA, USA). Sequence data were analyzed by using SeqScanner (https://sequence-scanner-software.software.informer.com/2.0/) and compared with published F8 cDNA (GenBank accession number NM_001003212.1) by using BLASTN (https://www.ncbi.nlm.nih.gov/).

### 2.4. Further Genetic Analysis

For genotyping the family members and 20 unrelated dogs we used fragment length analysis on an ABI Genetic Analyser 3130 (Life Technologies, Carlsbad, CA, USA) after amplification of the region containing the SINE insertion with FAM-marked primer (see Table S1). Data were analyzed by using GeneMapper (Life Technologies, Carlsbad, CA, USA). Five hundred and twenty-eight dog genomes available in the Dog Biomedical Variant Database Consortium (DBVDC) [12] were screened using bcftools view on the vcf file published by the DBVDC. The genomes originated from 126 different breeds and included 4 Rhodesian Ridgebacks.

## 3. Results

### 3.1. Clinical Presentation (Case Report)

An 8-week-old intact male Rhodesian Ridgeback puppy (A) was presented to the University Animal Hospital at the Norwegian University of Life Sciences with acute onset 5 out of 5 degrees lameness located in the right stifle joint. There was no prior history of trauma. Physical examination was otherwise unremarkable. Radiographs showed soft tissue swelling in the area. Despite conservative treatment with opioid analgesia, non-steroidal anti-inflammatory drugs, and rest, the affected joint showed waxing and waning symptoms. Soon additional joints were involved. The owner also reported continuous bleeding after teething. Cytology from joint taps was consistent with bleeding. Biochemistry was unremarkable, but the hematology showed mild neutrophilia and moderate regenerative anemia. An initial coagulation test showed prolongation of activated partial thromboplastin time and normal prothrombin, suggestive of a secondary coagulopathy affecting the intrinsic pathway. Factor IX activity was mildly reduced, but not interpreted to cause such severe clinical signs. An extended coagulation profile reviled severe reduction in Factor VIII activity and a diagnosis of Hemophilia A was made (Table 1). This finding sparked a retrospective investigation of the clinical history of the male siblings. The dog was euthanized at the age of 9 months due to poor animal welfare after repeated severe intra-articular bleedings.

One male sibling (B) had a history of acute onset coughing and severe dyspnea, without hyperthermia, at 8 weeks of age. The condition showed rapid (hours) improvement after stabilization with oxygen and broad-spectrum antibiosis. Retrospectively, a pulmonal hemorrhage was deemed possible. At 12 weeks of age, the dog developed acute 5 out of 5 degrees hind limb lameness located to the stifle joint. A severe grade patella luxation was also found. The dog was scheduled for surgical correction, but the surgery was postponed when alerted about a possible bleeding disorder. An intraarticular bleeding and hemophilia A were then confirmed (Table 1). The surgery was canceled and the dog has repeated incidents of lameness. It is still alive (age 1 year and 2 months).

A third sibling (C) was euthanized prior to this study due to severe post-surgical bleeding of a soft tissue swelling at the age of 11 weeks (Table 1). Retrospectively, hemophilia A was the likely cause of excessive bleeding. The remaining siblings were found to be healthy based on clinical examinations and/or history. 

### 3.2. Genetic Analysis

**Figure 1:**
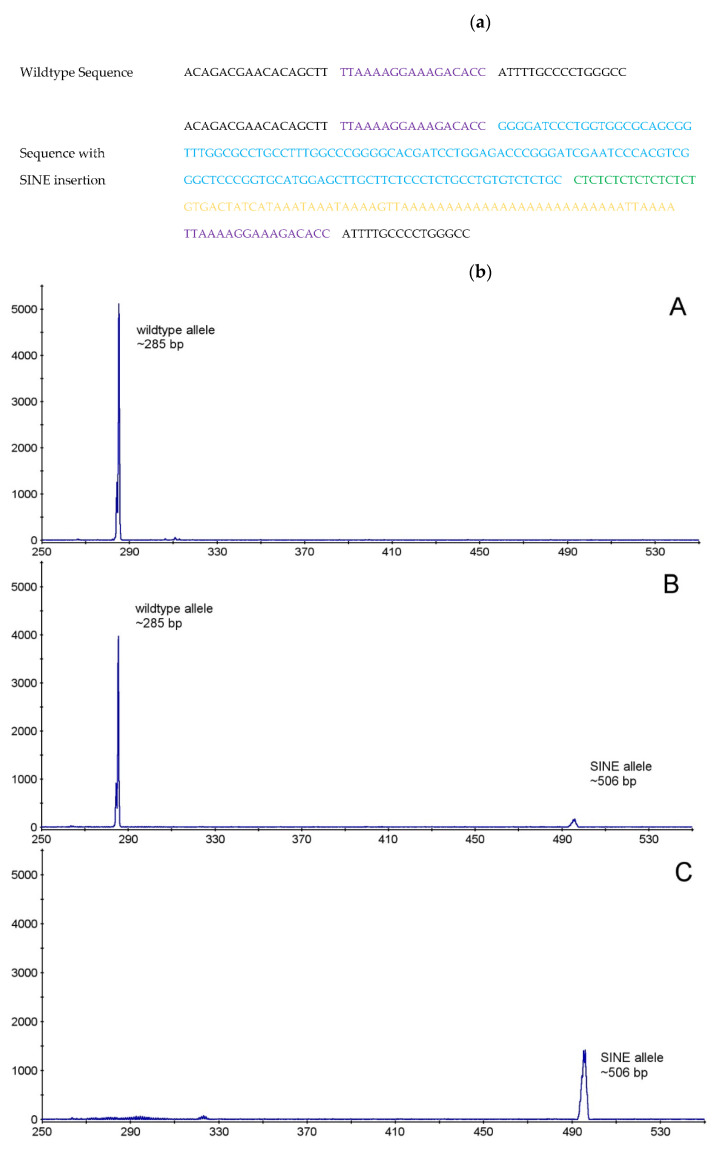
(**a**) Nucleotide sequence of the c.4824_25ins221 variant in exon 14 of *F8* gene of affected dogs. Black normal wildtype sequence, violet direct repeat, blue short interspersed nucleotide insertion (SINE), green (CT)_8_ stretch, yellow poly(A) tract (**b**) Fragment length polymorphism analysis of a wildtype (lane A), carrier (lane B) and affected (lane C) dog. Expected PCR product length without SINE 285 bp, with SINE ~506 bp.

First, the *F8* gene of the two affected males was investigated by cycle-sequencing all 26 exons with adjacent exon-intron boundaries in order to find the causal variant. By comparison with published cDNA sequence (GenBank accession number NM_001003212.1) one nucleotide exchange was detected. This variant is listed in ensembl (transcript F8-201, ENSCAFT00000083478.1) as synonymous SNP rs852889925. Furthermore, an insertion in exon 14 at position 4824 was observed. The insertion consisted of a direct repeat of the 17 flanking nucleotides, a tRNA-derived SINE, a (CT)_8_ stretch, and a poly(A)-tract. It was about 221 bp in length, and slightly variable in length due to variable length of the poly(A)-tract (Figure 1).

As the variant c.4824_25ins221 extended the PCR product of the exon 14 fragment of the F8 gene, further analysis of family members was made by fragment length polymorphism analysis (Figure 1). These analyses showed a perfect correlation of the variant with clinical signs of disease in the pedigree and a perfect segregation between affected and unaffected dogs (Table 2). Both affected male dogs carried the SINE insertion hemizygously, the mother and 4 female siblings were heterozygotes, while all three unaffected male dogs (father and two male puppies) and five female puppies did not carry the variant. One dog from the same household, but unrelated to the investigated family, showed the wildtype genotype. Samples of further relatives were not available.

When investigating the genotypes at the SINE insertion site of the cohort of 20 random Rhodesian Ridgebacks by fragment length polymorphism analysis, all dogs carried only the wildtype allele. 528 dog genomes (from 126 breeds) available in the Dog Biomedical Variant Database Consortium (DBVDC) [12] were screened for the presence of an insertion at the position of the found SINE (chrX: 1240738676_77) using bcftools view on the vcf file published by the DBVDC. Neither the four listed Rhodesian Ridgebacks nor the other dogs showed an indel at the position of the identified SINE variant (chrX: 1240738676_77).

## 4. Discussion

In this study, we identified a structural variant in exon 14 of the *F8* gene as a probable genetic cause of hemophilia A in a family of Rhodesian Ridgebacks. The variant was characterized as c.4824_25ins221 consisting of a 17 bp duplication, a tRNA-derived SINE, a (CT)_8_ stretch, and a poly(A) tract. It was found in the mother, both of the clinically affected male offspring and approximately half of the female offspring. The two affected males showed the variant hemizygously, whereas the mother and four additional female puppies were heterozygotes. All unaffected males and five female siblings showed only the wildtype allele. Hence, the occurrence of the SINE variant in the family tree showed an inherence pattern typical of X-linked recessive traits. Additionally, this variant was not found in other unrelated dogs of the same breed or dogs of other breeds. Therefore, the SINE insertion is not a common finding, and therefore deemed clinically important.

The other so far identified genetic variants in dogs carrying or suffering from hemophilia A lie further upstream of the SINE c.4824_25ins221; Pro471Arg in Boxers [8], Cys548Tyr in a German Shepherd [8], Arg577* in Old English sheepdog [7], and Trp33* in a German Shepherd [9]. These four variants are associated with a severe form of Hemophilia A with <1% FVIII activity. Additionally, there is a SINE (GenBank accession number *HE574814*) described as a cause of hemophilia A in Havanese dogs (which is patented) [10]. This SINE is also inserted in exon 14 of the *F8* gene, but about 800 nucleotides upstream, and shows 90,27% homology to the SINE c.4824_25ins221 in the investigated Rhodesian Ridgeback family. In humans, 2015 unique *F8* variants are listed in the F8 Variant Database (http://www.factorviii-db.org/) with a portion of 1.7% being insertions. *LINE*-1 and *Alu*-insertions in exon 14 of the human *F8* gene are known to be the cause of Hemophilia A [13,14,15]. In addition, an insertion with a length of 280 bp in exon 14 is listed in the database of the Institute of Human Genetics University of Wuerzburg (personal communication of Dr. Hasenmueller).

Short interspersed nuclear elements are described as a genetic cause in several other diseases in dogs. In Labrador Retrievers, a SINE insertion in exon 2 of the *PTPLA* gene is described to cause centronuclear myopathy. This tRNA-derived SINE probably has several effects on the mature protein: transcription of the whole SINE transcript or parts of it together with the normal mRNA, and thereby production of mutated protein, exon-skipping in the pre-mRNA involving several exons or the reducing of the *PTPLA* transcripts amount [16]. In Belgian Shepherds, a SINE insertion in exon 2 of the *ATP1A2* gene probably causes spongy degeneration with cerebellar ataxia (SDCA2) by complete skipping of exon 2 or activation of cryptic splice sites [17]. The same mechanism is supposed to cause Warburg micro syndrome 1 (WARBM1) in Alaskan Huskies. A SINE insertion of 218 bp length is inserted in exon 7 of *RAB3GAP1* gene introducing a new splice site and leading to the formation of a novel mRNA containing 187 nucleotides instead of the exon 7 sequence [18]. Similarly, early retinal degeneration cosegregates with an exonic SINE insertion in exon 4 of *STK38*L gene causing loss of the N terminus of the translated protein [19].

Unfortunately, we were not able to investigate mRNA of the affected males. But the description of SINE insertions as a genetic cause in other diseases is a strong argument for this SINE variant damaging the functionality of the FVIII protein. The SINE variant may either cause altered splicing and subsequently building of defect FVIII, or it may be translated along with the original exon 14 leading to the introduction of stop codons and production of a shortened and probably unfunctional FVIII protein. Both the altered splicing and the altered translation would lead to a protein with a mutated or missing light chain. The light chain contains the A3, C1, and C2 domains encoded by exons 14 to 26. The C2 domain is described as essential for FVIII-vWF complex formation which enables the circulation of FVIII in the bloodstream [20]. Hence, the lack or alteration of the light chain probably leads to the early decay of FVIII and undetectable FVIII activity.

The first described variant in dogs with Hemophilia A was the intron 22 inversion in the Chapel Hill colony and later in the Queen´s University colony. It resembles the most common human mutation in the *F8* gene [5,6]. This inversion leads to the production of mRNA where the exons 23 to 26 are replaced by a novel sequence, and subsequently production of an FVIII protein lacking the C2 domain. Hemophilia A in dogs has been described in different grades of disease. As the colony dogs, our dogs also suffered from a severe form of Hemophilia A, also supporting possible common involvement of a defect C2 domain.

In summary, the identified SINE insertion in exon 14 and subsequent alteration of the light chain of the FVIII protein probably is the underlying genetic cause of Hemophilia A in the studied family of Rhodesian Ridgebacks. The SINE c.4824_25ins221 correlates perfectly with the signs of disease and an X-linked recessive trait in the family tree, and it is not present in unrelated dogs of the same and other breeds. Furthermore, SINEs are the cause of other inherited diseases in dogs, hemophilia A in humans and even in another dog breed. In addition, the alteration of the light chain of the *F8* gene by the intron 22 inversion (which is similar to the alteration probably caused by the SINE) is the underlying cause of a severe form of canine and human hemophilia A.

Our results enable genetic testing of Rhodesian Ridgebacks lines where Hemophilia A is suspicious because of affected male offspring in previous matings or relationship to the studied family. The five identified female carriers should be excluded from breeding and the female siblings of the mother should be genotyped before breeding to avoid producing clinically affected male puppies who have to be eventually euthanized. The prevalence of the variant in the whole breed is yet unknown and the possibility that the described variant is a private mutation cannot be ruled out. Nevertheless, a genetic test can be a tool for veterinarians and breeders to confirm or exclude the existence of the described variant in suspicious and breeding dogs. Once confirmed that a dog with signs of Hemophilia A carries the SINE insertion, the identification of and breeding with female siblings carrying only the wildtype allele is possible.

## Figures and Tables

**Table 1:** Summary of clinical signs and clinicopathological parameters in affected dogs.

Parameter	Puppy A	Puppy B	Puppy C	Reference Range
Prothrombin time	8.1 s	-	-	<10.1 s
Activated partial thromboplastin time	25.9 s	-	-	<13.5 s
Thrombin clotting time	10.3 s	-	-	<18 s
Fibrinogen	1.9 g/L	-	-	1.2–2.9 g/L
Factor IX activity	59%	-	-	75–140%
Factor VIII (activity)	2% (severe Hemophilia A)	2% (severe Hemophilia A)	-	70–125%
Von Willebrand factor (antigen)	171	-	-	>70
Clinical history and signs	Prolonged bleeding from umbilical cord. Repeated severe intra-articular bleeding. Continuous post-teething bleeding. Euthanized due to animal welfare.	Prolonged bleeding from umbilical cord. Repeated severe intra-articular bleeding. Possible pulmonary bleedings (retrospective findings). Alive.	Prolonged bleeding from umbilical cord. Massive soft tissue bleeding after possible trauma to head. Euthanasia due to post-operative uncontrolled bleedings.	

**Table 2:** Phenotype and genotype distribution in study dogs.

Dog	Signalement	Pedigree	Clinical Signs ^a^	Genotype c.4824_25ins221
1	female, * 28.03.15	Mother of 3–15	-	X(n)/X(ins) (carrier)
2	male, * 24.07.16	Father of 3–15	-	X(n)/Y (normal)
3	Male (A), * 07.10.19		**+**	X(ins)/Y (hemizygous affected)
4	Male (B), * 07.10.19		**+**	X(ins)/Y (hemizygous affected)
5	male, * 07.10.19		-	X(n)/Y (normal)
6	male, * 07.10.19		-	X(n)/Y (normal)
7	female, * 07.10.19		-	X(n)/X(ins) (carrier)
8	female, * 07.10.19	Puppies of 1 and 2	-	X(n)/X(ins) (carrier)
9	female, * 07.10.19		-	X(n)/X(ins) (carrier)
10	female, * 07.10.19		-	X(n)/X(ins) (carrier)
11	female, * 07.10.19		-	X(n)/X(n) (normal)
12	female, * 07.10.19		-	X(n)/X(n) (normal)
13	female, * 07.10.19		-	X(n)/X(n) (normal)
14	female, * 07.10.19		-	X(n)/X(n) (normal)
15	female, * 07.10.19		-	X(n)/X(n) (normal)
16	male, * 01.06.18	unrelated	-	X(n)/Y (normal)

^a^ clinical signs: See Table 1, * born at.

## Data Availability

Datas are available directly from the authors.

## Supplementary Materials

The following are available online at https://www.mdpi.com/2073-4425/12/2/134/s1, Table S1: Primer sequences.
