# Supplementary material for: A SINE Insertion in F8 Gene Leads to Severe Form of Hemophilia A in a Family of Rhodesian Ridgebacks
Source: Genes (Basel). 2021 Jan 21;12(2):134. doi: 10.3390/genes12020134 (PMC7909816; doi:10.3390/genes12020134)
Supplement: Supplementary file 1 [file genes-12-00134-s001.pdf]

Table S1

Primer Sequences for amplification and Sanger Sequencing of canine FVIII gene

Exon	forward	reverse
1	TTT CTC CTG GGAG GCT GAA GA	CCCTATGGAACACGCCTTTA
2	AAAAAGGGTTCACCAAAATAAATG	GGACATCAGTTACTCAGTTGCAT
3	ATCACAGGGGCATGTTTTTC	CCTGAGATGACAGGACAACG
4	TCAACAGTGGGTATGGAAAGG	CTCCCTTATTTCATTTGAGTCCT
5	TGATGTCTCCTAGTGTTGATTTCC	CCAAAGCAGATTGCAGTTCA
6	GTCCTCGCCTCCTCTTTCAT	GACAGAGTCCGAGGCTAACG
7	GGTCAATTTGTCCATCCTATCC	CATTCAAGGTGAAATCTTATACTGG
8	CCCTAACATTGTTTGTTTTGTCC	CACGGCTTGAGAATTTGTTG
9	TTCTCCATCCCAACATCTCA	ACTCAACCTTACTTCTCTTCTTCAA
10	AAC AAA CCC CTG TGA TAC GG	GGG TCA GGA CCA CTA CGA T
11	CGT AAA GCC CTT GAA ATA ACA AC	CTG CAG GCT ATA GGG GGA TG
12	CGTGCCATCACTTCCATGT	TTGGTGGCTTCAATTTTCTTTT
13	TCT TCT TGG GAA TAA GGT AAT GG	TCA CCC TCT GCC ACT CTC TC
14	GGGACCAGGGTTGTGAGTAA	GTCCCAACAGCATCAACAAA
14	CAATCTGGAGAAAGAACACAGC	GCAGTTCTGGCTCAGGAGTAA
14	CCGTCACAGTGAGGACAGAG	AAATACAATGGTACCTAAATGACTG
14	CCCCCAAATATGTCAGTTCA	CATTATCTTTGATTAATGAAGCAGG
14	TGAGAGAAAATGTATTATCAATGGA	GCTCTAGTATTTCTGTCCATAAACG
14	CAGTACCTCAGTCTGGCAAGATA	ACTGGGCCTTTGCTCAGA
14	GGATAAAGACCCATGGCAAG	TTCTTTCTATCTCTTCCGGAGATT
14	TTGCTAACTTGGCTAATGTCCA	CCCTTCACTATGTGGCGAAT
14	CAGGTTTAGAAGAGCAGCCATA	CATTTGCAATAACCTTCCTTTCA
14	AACGTGGTAAGCGGAGTTG	TTTGCAACGGGTAATGCAG
14	GAGGAATCATGTCACCATTCAA	TGAACTTTCCTTGCCCTTCA
14	GAAATAACCTCTCTTTAGCCTTTG	TTTCCCATGAGATCCAGGT
14	TTTCCCTACAAAAACTAGCAATGA	AAAAGCTGTGTTCGTCTGTGA
14	TTGCTTGGGATAACCACTATGA	TTTGTCTTCCTCTGGCTGAA
14	GCTCTCAAAACCCACCAGTC	TCAGCACCTGAAGGCAGAG
15	TCA AAT ACA GGG AGG GCA AG	AAG AGA ACC TAC ACC GAA ATG G
16	AAAAGACACTTGAATTTTCTGTAAA	CCAGTAAGTGGTCAGAGCAATG
17	GGGATTGCTGTCTTCTCTCC	GAGGCTCCCTTGCTGAGG
18	TCAACAGCCTTTTCTGTCTTCTC	GAGGTGGAAGAGGGCACA
19	CGTATCTCATGCTCATTGCTTT	AGAAAGGCAACCATGCTAGGA
20	CTGTCGTCCTCCCCTCCT	TCAGCTCAGTTCTTCGAGACA
21	CCA ACG CAG CTG AAT CTA ATC	TGT TTT GAG CTT GCA AGA GG
22	CAG GTC CTT CTC AAG CCA AC	TCC CGA ATT TGA AAT CTT TGT C
23	CCTGGCATGTACCTGTGCTA	GGAAGGGCACGTGATGACT
24	GGC TGC CTC TTG TCC TTA TG	CTG CTC TGC GTC GGT TAA G
25	CGG TAC TGT TCC CCA GGA TAC	GAG AGG TTT TGT GCC TTT CC
26	CCT GGA AGC TGC TGG AAG	CAG GAC CTC CAG CCT CAG

Primer for Fragment lenght polymorphism analysis

14	FAM-TTGCTTGGGATAACCACTATGA	TTTGTCTTCCTCTGGCTGAA
